# The prevention and management of postoperative trachomatous trichiasis: A systematic review

**DOI:** 10.1016/j.survophthal.2023.02.008

**Published:** 2023-03-05

**Authors:** Andreas J. Kreis, Emily W. Gower, Martina Kropp, Amir B. Kello, Guirou Nouhoum, Serge Resnikoff, Sandra L. Talero, Anthony W. Solomon

**Affiliations:** a Experimental Ophthalmology, University of Geneva, Geneva, Switzerland; b Department of Ophthalmology, University Hospitals of Geneva, Geneva, Switzerland; c Gillings School of Global Public Health, University of North Carolina at Chapel Hill, Chapel Hill, NC, USA; d World Health Organization Regional Office for Africa, Brazzaville, Congo; e Techniques and Technologies of Bamako, Institut d’Ophtalmologie Tropicale d’Afrique, University of the Sciences, Bamako, Mali; f Organisation pour la Prévention de la Cécité, Paris, France; g School of Optometry & Vision Science (SOVS), University of New South Wales, Sydney, Australia; h Research Department of Development and Innovation, Superior School of Ophthalmology, Barraquer Institute of America, Bogotá, Colombia; i Department of Control of Neglected Tropical Diseases, World Health Organization, Geneva, Switzerland

**Keywords:** Postoperative Trachomatous, Trichiasis, PTT, Trichiasis Surgery, Trachoma, Systematic Review

## Abstract

Among ocular infections, trachoma is the main cause of blindness. Repeated conjunctival *Chlamydia trachomatis* infections lead to trichiasis, corneal opacification, and visual impairment. Surgery is often needed to relieve discomfort and preserve vision; however, a high postoperative trachomatous trichiasis (PTT) rate has been observed in various settings. We wanted to know why, whether PTT rates could be reduced, and how to manage the PTT that occurs. We performed a search of the literature. Of 217 papers screened, 59 studies were identified for inclusion as potentially relevant, the majority having been excluded for not directly concerning PTT in humans. Preventing PTT is a major challenge. Only one published trial, the STAR trial in Ethiopia, has reported a cumulative PTT rate <10% one year after surgery. The literature on the management of PTT is sparse. Though no PTT management guidelines are available, high-quality surgery with a low rate of unfavorable outcomes for PTT patients is likely to require enhanced training of a smaller group of highly-skilled

## 1 Introduction

Trachoma is a disease caused by particular strains of *Chlamydia trachomatis*^1^ that are primarily characterized by typing of the major outer membrane protein. Serovars A, B, Ba, and C associate with trachoma.^2^ A public health problem in parts of at least 42 countries,^3^ it is concentrated in low-income communities and, within those communities, tends to affect the poorest.^4^ It is the leading infectious cause of blindness. About 1.3 million people are irreversibly blind from trachoma.^5^

**Figure 1:**
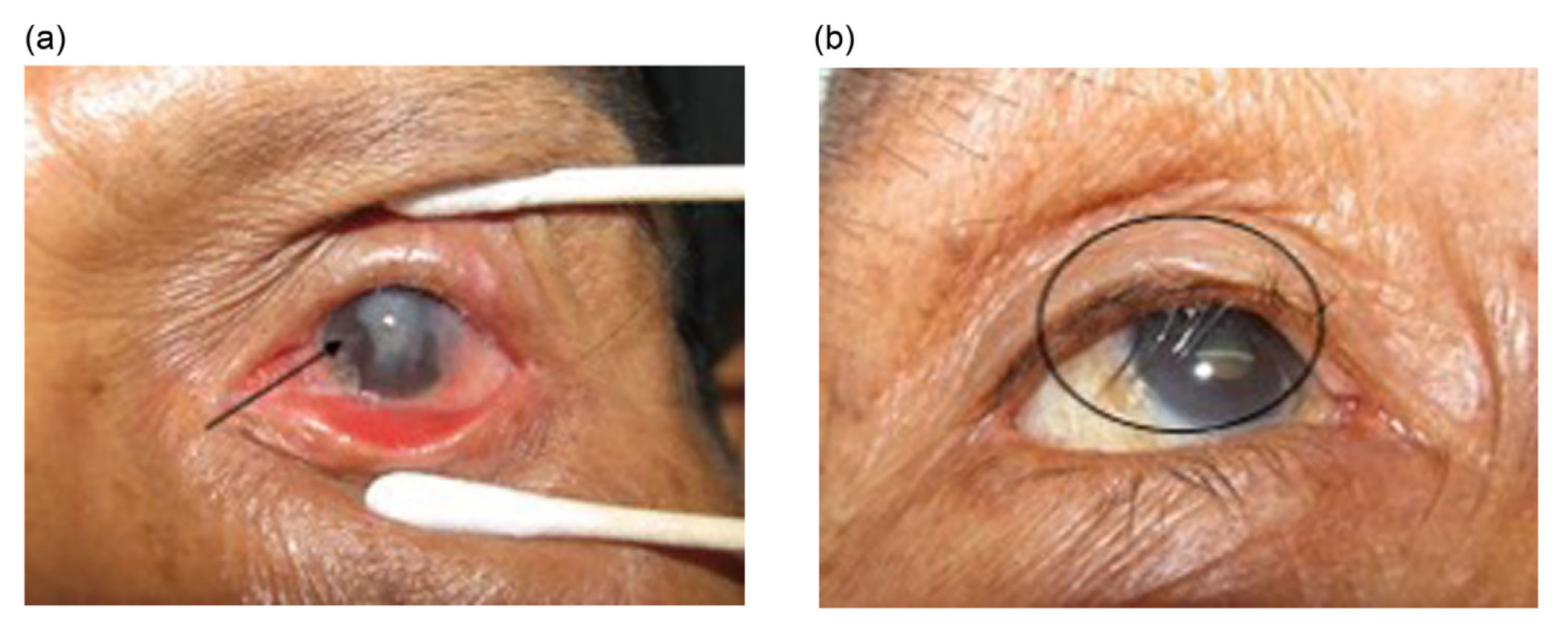
Two patients with trachomatous trichiasis (circled) and concomitant corneal opacities (arrow).

Ocular *C. trachomatis* is thought to be transmitted relatively easily in trachoma-endemic communities, particularly among household contacts^6–8^ and, therefore, recurs often. With repeated infections, infection-induced scarring leads to entropion and trachomatous trichiasis (eyelash inversion or TT), corneal scarring, and eventually to blindness (Fig. 1).^9,10^ In June, 2022, an estimated 1.7 million people worldwide had TT.^3^ For pain relief and preservation of remaining vision, eyelid surgery for TT is a key component of the World Health Organization (WHO)-recommended SAFE strategy (surgery, antibiotics, facial cleanliness and environmental improvement).^11^

Numerous surgical techniques for managing TT have been described. Unfortunately, the rate of postoperative trachomatous trichiasis (PTT) after these procedures is high in many settings, with a cumulative incidence by 1 year after surgery ranging from 7 to 69%.^12–22^ The reasons for this range of PTT rates almost certainly relate to local variation in both imperfections in surgical technique and ongoing disease-related scarring processes.^23–29^ The relative contributions of these factors, and those of other potential stimuli, remain unclear.

Here we review the published literature on PTT with the aim of better understanding this condition. We try to address 2 questions: first, what is known about its pathogenesis and presentation? Second, what evidence is there for preventing and managing it?

## 2 Methods

### 2.1 Overall approach

A systematic review of papers relating to PTT was undertaken following PRISMA guidelines (http://www.prisma-statement.org). Full inclusion and exclusion criteria are outlined in Table 1. We prospectively registered the review on PROSPERO (https://www.nihr.ac.uk/166829), after searching the PROSPERO database (using the search terms nominated below) to prevent duplication of an existing, but as yet unpublished, review.

### 2.2 Search method

We searched PubMed (https://pubmed.ncbi.nlm.nih.gov/) without limits on the year or language of publication, including papers indexed in that database up to and including August 1, 2022. We used the search terms (“recurrent” OR “postoperative” OR “post-operative” OR “post-surgical” OR “postsurgical” OR “secondary”) AND “trichiasis.”

### 2.3 Data extraction

Two reviewers (AJK, AWS) independently screened the titles and (where available) the abstracts of the search output. Full-text versions of papers selected by either reviewer were obtained for more detailed scrutiny. Data extraction and study quality evaluation were performed independently by the same 2 reviewers. Reference lists of full-text papers were hand-searched for additional pertinent published research. All potentially relevant non-English publications were translated into English.

### 2.4 Data analysis

Information containing definition, pathophysiology, clinic features, epidemiology, treatment and prevention of PTT was compared across studies to identify similarities for cross-study comparison. Where there was a disagreement on whether or not to include a particular paper, the reviewers evaluated it together and reached agreement through discussion.

## 3 Results

### 3.1 Study eligibility

A total of 218 papers identified in the PubMed search; 128 were considered sufficiently likely to be relevant to warrant full-text review (Table 1). The remaining 90 papers were excluded for being in animals other than humans (6 papers) or covering oculoplastic in general and/or preclinical experimental studies (84 papers). Of the 128 papers assessed by full-text review, 68 were further excluded for not being directly related to PTT, but rather focusing on unoperated TT or trachoma in general. This left 60 papers for inclusion in our review. No papers describing PTT management were sufficiently methodologically sound to warrant formal assessment of the risk of bias or a meta-analysis. Instead, we conducted a qualitative synthesis of the 60 included papers.

### 3.2 Definition of PTT

PTT is defined by the presence of one or more eyelashes touching the eye, or evidence of epilation of in-turned eyelashes, after eyelid surgery for TT.^18^

### 3.3 Pathophysiology of PTT

Much is known about the pathophysiology of trachoma in general. In contrast, although many clinical trials of the management of primary TT use the incidence of PTT as an outcome measure, there are no structured studies of its pathophysiology, other than by Burton and coworkers, who suggest recurrent trachomatous trichiasis is associated with increased conjunctival expression of S100A7 (psoriasin).^23^ This lack of pathophysiological investigation is troubling, given the apparent high incidence of PTT documented in clinical trials and small cohorts. It has been argued that ongoing recurrent conjunctival *C. trachomatis* infection, with accompanying scarring as each infection resolves, might account for a proportion of the incidence.^22,29,30^ Additionally, an inflammatory process in the absence of further chlamydial infection can promote abnormal healing, leading to malposition of the eyelid and eventually PTT.^23,31^ It has also been shown that the location of trichiatic eyelashes preoperatively predicts the likelihood of developing PTT, with peripheral TT being more likely to be followed by PTT.^14^ Among other possible mechanisms (suboptimal surgical incision height, insufficient rotation, poor eyelid contour^16,32,33^), this association could potentially be explained by the fact that inexperienced, insufficiently dextrous, or apprehensive surgeons find it difficult to extend the eyelid incision as far temporally and nasally as is required to ensure adequate external rotation of the entire eyelid margin.^27,32^

In general, one could postulate that all the primary pathophysiological processes that culminate in incident TT could play important roles in post-surgical failure, leading to PTT.^14,16,23,34^ This is important, considering that scarring, in general, can be unpredictable, and scarred tissue is more difficult to operate on.^25^ Surgery itself does nothing to ameliorate the scarring process that underlies the development of TT and may in fact augment it. Undertaking surgery inappropriately (e.g., implementing an entropion correction procedure in the absence of entropion ^35^ or even implementing an entropion correction procedure in the absence of trichiasis ^36^) may, therefore, potentially also contribute to the overall PTT burden.

### 3.4 Classification of PTT

**Figure 2:**
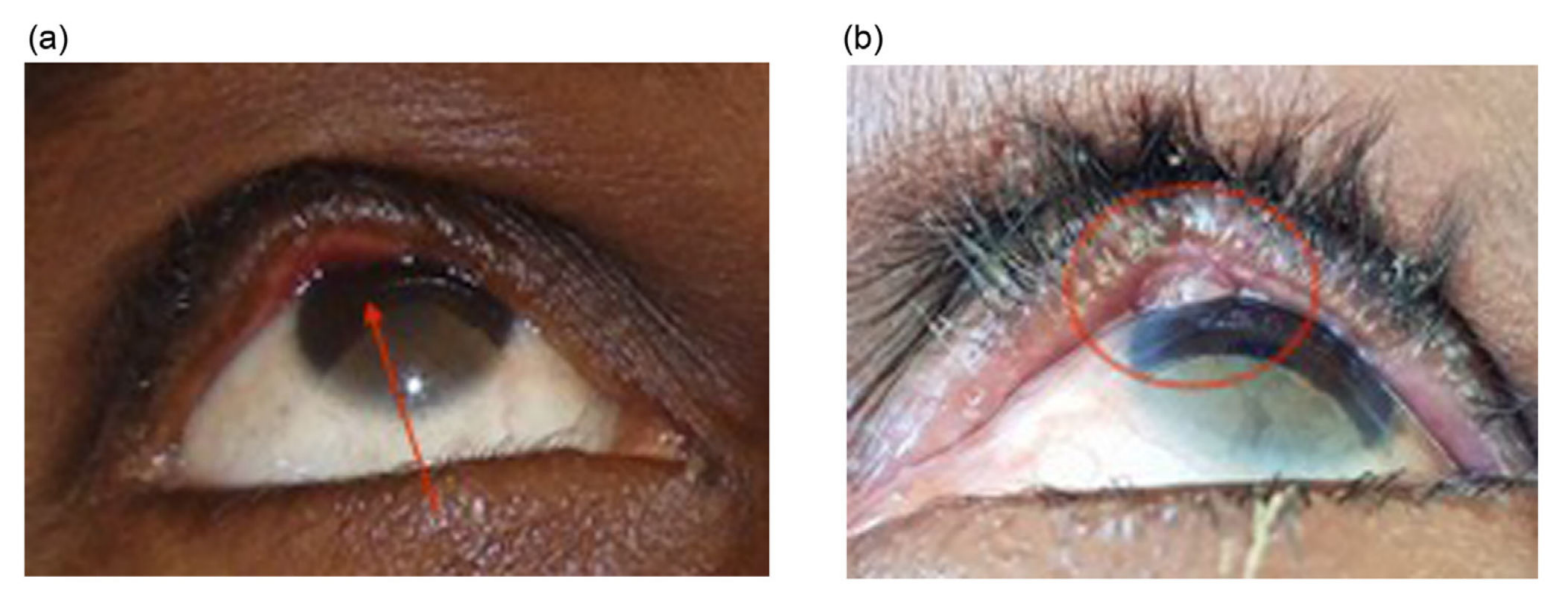
Eyelid contour abnormality (arrow) and pyogenic granuloma (circled) after surgery for trachomatous trichiasis.

The clinical features of primary TT are well known and have been described previously.^37^ The same features are found in PTT. Severity of PTT (Table 2) has been investigated and classified within the context of several trials.^14,29,33,38,39^ Other features complicating the original operation, including pyogenic granulomata and eyelid contour abnormalities (ECAs) (Fig. 2), may be found at the time of patient presentation with PTT.^16,24,32,40^ These additional elements may complicate the PTT management algorithm.

### 3.5 Epidemiology of PTT (incidence and risk factors)

Despite ongoing extensive efforts to eliminate trachoma globally, not enough is known about the epidemiology of PTT outside of clinical trials and other research settings.^41^ Surgical programs have reported unacceptably high PTT incidence, varying from 2 to 69% by 3–6 weeks after surgery and 7–41% at 1 year.^18,19,42–44^ Some research has focused on reducing the risk of PTT.^16,24,29,32,40,45^ At least 4 research groups postulate that more highly-skilled trichiasis surgeons can reduce the risk.^13,17,25,32,34,46,47^ It is also often assumed that risk factors that associate with primary TT, such as repeated conjunctival *C. trachomatis* infection and recurrent or ongoing chronic conjunctival inflammation, contribute to the risk of PTT too.^29,42^ In addition, system-related factors intrinsic to the patient pathway, such as aberrant patient selection for surgery, routine implementation of inappropriate technique such as inadequate rotation, and poor operating site infrastructure likely underlie high PTT incidence.^14,25,33^

### 3.6 Management of PTT

The aims of treatment for TT are to (1) prevent further corneal opacification due to trauma from the eyelashes abrading the cornea; (2) reduce the risk of ongoing corneal trauma providing a portal of entry for secondary corneal infection by bacteria or fungi; (3) relieve pain; and (4) recover some vision through relief of blepharospasm and reduction in corneal edema.^48^ These aims can be realized by repositioning the eyelashes so that they no longer touch the eye. For primary TT, one of 3 well-established procedures is usually used: bilamellar tarsal rotation (BLTR, Fig. 3A), a modification of Trabut’s tarsotomy (Fig. 3B), or tarsal advance and rotation (Fig. 3C). A range of other non-surgical and surgical treatments (Table 3) have been described for primary TT; these include epilation, eyelid taping, electrolysis of involved eyelash follicles, cryotherapy to involved eyelash follicles, excision of eyelash-bearing tissue (through, e.g., wedge excision), tarsoconjunctival grafts and flaps or some combination of these approaches, with or without upper eyelid blepharoplasty.^13,21,25,26,39,45,49–59^ It is likely that all of these approaches designed for the management of primary TT are also used in the management of PTT, though published data on this are sparse.

Anecdotally, the choice of treatment for PTT generally depends on factors such as available resources and expertise, location of the patient (which may influence the scope for follow-up), and disease severity, as well as providers’ impressions of global and local recommendations.

In most jurisdictions, eyelid surgery is delivered by trained ophthalmic assistants or ophthalmic nurses. In some settings, surgery is provided by ophthalmologists. Ophthalmologists generally perform only a fraction of the required operations because there are too few of them to meet the surgical demand, and those that exist are concentrated in major urban areas, limiting the ability of patients with TT or PTT to access the service they provide.^13,61–63^

We found no published guidelines on the management of PTT.^25^ Although epilation has been found to be an efficacious alternative to surgical management of minor unoperated and postoperative TT cases,^14,15^ surgery to correct the eyelid deformity seems to be widely recognized as the treatment of choice for PTT.^25^ Anecdotally, approaches have evolved over time ^64^ and 2 recent publications have described novel methods addressing PTT.^65,60^ The feasibility at programmatic level, however, needs to be discussed.^46^

Over 60% of primary TT cases and, therefore, probably also most cases of PTT, occur in sub-Saharan Africa.^9^ In most African countries affected by trachoma, primary TT is generally managed by ophthalmic nurses who have limited surgical training.^13^ PTT, a significantly more complex surgical problem, is typically managed using the same techniques and by the same personnel who undertake primary TT surgery. WHO, while noting the need for more research on the treatment of PTT, proposes that in the absence of specific evidence on optimal approaches, it “should be managed by the most experienced trichiasis surgeon or eye specialist available” and that “between diagnosis and review by that professional, epilation should be encouraged.”^66^

### 3.7 Prevention of PTT

Two randomized clinical trials indicate that perioperative single-dose oral azithromycin can help prevent PTT, while a third trial failed to show a benefit of azithromycin in a setting where lower surgical quality led to high PTT rates.^21,67–70^ Two other studies cast doubt on the efficacy of azithromycin for this indication.^13,70^ A randomized trial in Ethiopia comparing 28 days of adjunctive doxycycline to placebo showed no difference in the cumulative incidence of PTT at month 12 and a much higher incidence of adverse events in the doxycycline group.^17^ A recent pilot study suggests that fluorometholone 0.1% may be safe and efficacious in reducing PTT incidence following initial TT surgery, with 1 drop twice daily for 4 weeks identified as the most promising dose.^71^ Given the diversity of outcomes, further long-term data to determine the value of oral or topical azithromycin, other antibiotics, and topical fluorometholone are needed.

We found no published data demonstrating management approaches for primary TT that reduce the risk of PTT to a point at which it could be considered a rare event: the lowest reported incidence of PTT at 1 year after surgery is 7%;^72^ however, at least seven major studies and reports have suggested that PTT is in part attributable to suboptimal surgical skill or performance.^14,22,27,32–34,46,47^ A randomized, controlled trial conducted in Ethiopia with 1,200 patients suggested that the incidence of PTT is lower following a modified Trabut procedure compared to BLTR.^16^ A much smaller trial (18 patients) did not demonstrate a difference in PTT incidence comparing BLTR with simple anterior lamella rotation (ALR); completion of a larger randomized study comparing these interventions was suggested.^49^

Apart from incision height and degree of rotation of the marginal eyelid fragment, exactly which aspects of primary TT surgery most influence outcome are unclear.^33^ It seems intuitively likely that, in addition to identifying surgical protocol amendments that decrease PTT incidence (potentially including adjunctive steroids or antibiotics, or alterations in details of the recommended surgical technique), there is a need to improve the level of surgical skill and increase the reliability of postoperative follow-up strategies and systems. We found no studies evaluating surgical set up, including theatre lighting, surgical supervision, routine audit, follow-up systems, or monitoring of PTT. Similarly, we found no studies comparing the use of magnifying loupes by eyecare workers versus none during surgical procedures of TT or PTT surgery.

## 4 Discussion

Poor outcomes from TT surgery affect both the individual patient and the trachoma elimination program as a whole, since confidence in surgery is likely to affect community confidence in the worth of other components of the SAFE strategy. A good understanding of the factors that increase the likelihood of an adverse outcome is, therefore, crucial for surgeons, surgical trainers, and program planners.^16,25^

Several factors have been linked to the high incidence of PTT. The strength of evidence for these associations is variable, and it is presently impossible to propose a universal way in which the incidence of PTT could be expected to be reduced sufficiently such that patients operated on for primary TT would almost invariably advocate for the operation. Though robust evidence is lacking, there are frequent calls in the literature for strengthening of TT surgical systems. Requested measures include improvements in the physical infrastructure, like adequate lightening, operating table and chairs, and surgical equipment, upon which TT surgery is dependent; the skills of and robustness of certification for TT surgeons; and the systems that facilitate follow-up, routine audits and surgical supervision.^22,25,27,32–34,47,73^ We found no studies that addressed the impact of these factors. Empirically examining their impact should be considered in future work.

Given the lack of hard data identified in our systematic review, we offer some general thoughts flavored by the papers we identified, but based mostly on our own experiences and the first principles of surgical management. Imperfect lighting, lack of cleaning services, suboptimal operating tables, and low quality cautery devices make surgery considerably more challenging.^25^ From the surgeon’s viewpoint, such deficiencies add technical barriers, slow patient throughput, and heighten team and patient stress. Eyelid surgery in general is delicate work, requiring dexterity and a clear, magnified field of view; it is even more difficult in the already-operated eyelid with PTT. A significant proportion of primary TT surgeries, however, are undertaken in outreach settings^62,74^ where the fixed infrastructure for operating is likely highly variable. Studies reporting no difference in outcomes between surgery performed in rural villages and in the hospital setting are testament to the skill of the surgeons involved.^75^ Such skill is even more critical in PTT, where patient and provider have to deal with consequences of previous failure. To give surgery the best possible chance of success, adequate infrastructure, working equipment, and the availability of binocular magnification are necessary (but probably not sufficient) to facilitate good outcomes.^25^ Further studies investigating, for example, the impact of systematic use of binocular loupes on surgical outcomes could be considered.

The roles of azithromycin, fluorometholone, and other antibiotics for reducing the incidence of PTT remain unclear and need to be further investigated.^71^ It is possible that more active patient tracking^73^ might allow ophthalmic assistants or ophthalmic nurses to identify abnormal healing or conjunctival inflammation at an early stage, with reactive introduction of steroids or antibiotics.

We found no published data to assist decision-making regarding which surgical technique should be preferred when managing PTT, although this question is currently being investigated in a randomized trial. Numerous approaches are currently used in practice and sometimes even combined, including procedures like wedge resections for trichiasis or ble-pharoplasty.^25^ A similar situation is seen in glaucoma management,^76^ but not in cataract surgery, where there is over-whelming evidence on how to best perform surgery, and the surgeon’s choice is generally the gold standard approach of phacoemulsification in high-income settings, or manual small incision cataract surgery in low income settings.^77–79^ We postulate that for both glaucoma and PTT, the existence of numerous management approaches is associated with poor outcomes and consequent loss of patient trust. Studies systematically looking at the combined use of multiple techniques for PTT versus the use of one of the standard techniques described above could be informative.

Intraoperative patient discomfort at the time of primary surgery could lead to reluctance to return for review or reoperation if needed, and poor uptake of primary TT surgery by others in the community.^80,81^ Intraoperative patient discomfort (through either pain or anxiety) at the time of surgery for PTT would make achieving good results extremely difficult. PTT by definition occurs in a previously operated eyelid, which therefore will be scarred and may also be inflamed. The volume of local anesthetic used must be sufficient to completely numb the tissues. Failure to do so puts both surgeon and patient under significant stress because of patient pain and potential movement.^25^ The possible roles of adjunctive hyaluronidase to improve local anesthetic tissue penetration and/or steroids to decrease postoperative tissue inflammation in PTT have not been fully explored.^82–85^ Training of surgeons in good anesthetic technique and provision of postoperative analgesics are both important.^86^

In the absence of better evidence, PTT management should be undertaken by highly skilled hands. This may need to be an oculoplastic surgeon, ophthalmologist or a specially-trained TT nurse, depending on the local context.^87^ Based on first principles for revision surgery at any anatomical location, individualized patient assessment and planning are needed. The complexity of the distorted anatomy and scarred and inflamed tissue require management by experts.^25,46^ To achieve this, we think that the patient pathway for PTT should be reconsidered and redesigned. Although ideally PTT patients would be directed to tertiary level hospitals designated as PTT centers where well-trained oculoplastic surgeons manage cases with support from a multidisciplinary team, this would not be not practical in the remote settings where most PTT patients live. Instead, programs may elect to choose particularly skilled TT nurses and invest in their training to generate a cohort of PTT nurse-surgeons.^18^

In any case, the selection and training processes for TT and PTT surgeons should be clearly defined and adhered to, acknowledging that training processes have evolved over time.^88–90^ Evaluation of TT surgeons themselves for uncorrected refractive error, cataract and inadequate stereoscopic vision should be a mandatory part of the selection process.^18,91^ Structured program monitoring and evaluation as well as further clinical research will be key to decreasing the complication rate.^33,92^ Finally, a no-fault error culture must be fostered in which it is recognized that untoward events occur despite the best efforts of all involved. This will facilitate ongoing learning, system improvement, enhanced patient safety and better outcomes.^93–95^

## 5 Conclusion

To date there are no management guidelines available for PTT and an incomplete understanding of PTT prevention. More clinical studies are needed to generate evidence. In the mean-time, TT surgical training needs to be intensified and further improved. Specific patient pathways for PTT need reconsideration. Infrastructure in general requires ongoing improvement.

### 5.1 Methods of literature search

We searched PubMed (https://pubmed.ncbi.nlm.nih.gov/) without limits on the year or language of publication, including papers indexed in that database up to and including August 1, 2022. We used the search terms (“recurrent” OR “postoperative” OR “post-operative” OR “post-surgical” OR “postsurgical” OR “secondary”) AND “trichiasis.”

Articles were independently screened for eligibility in two stage. First stage by the titles and (where available) the abstracts of the search output. Second stage, full-text versions of papers selected by either reviewer were obtained for more detailed scrutiny. Data extraction and study quality evaluation were performed independently by the same two reviewers. Reference lists of full-text papers were hand-searched for additional pertinent published research. All potentially relevant non-English publications were translated into English.

### 5.2 Disclaimer

ABK and AWS are staff members of the World Health Organization. The authors alone are responsible for the views expressed in this article and they do not necessarily represent the views, decisions or policies of the institutions with which they are affiliated.

## Figures and Tables

**Figure 3A:**
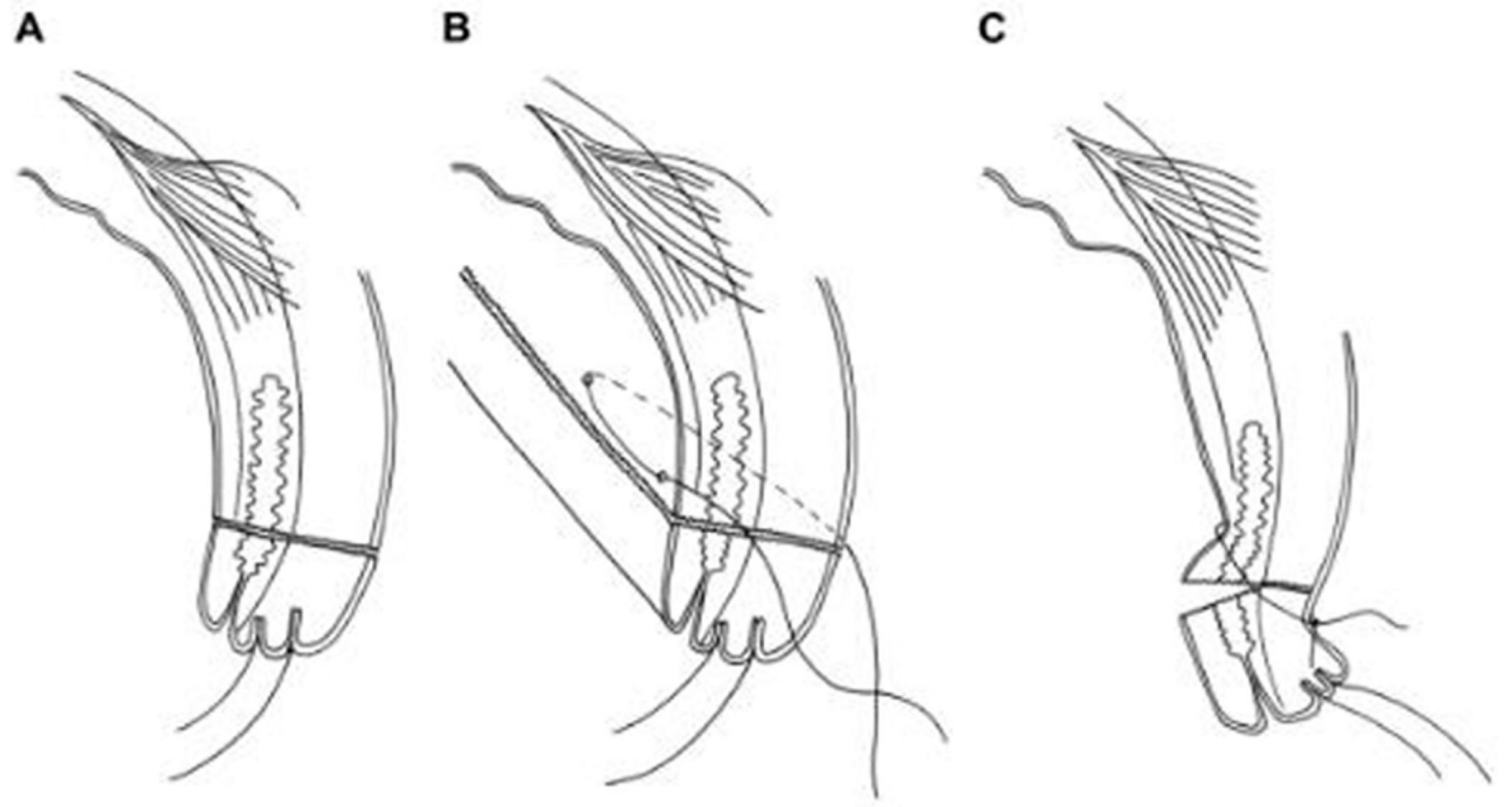
Bilamellar tarsal rotation^48^. *A*: Bilamellar incision. *B*: Horizontal mattress suture. *C*: Postoperative lid eversion.

**Figure 3B:**
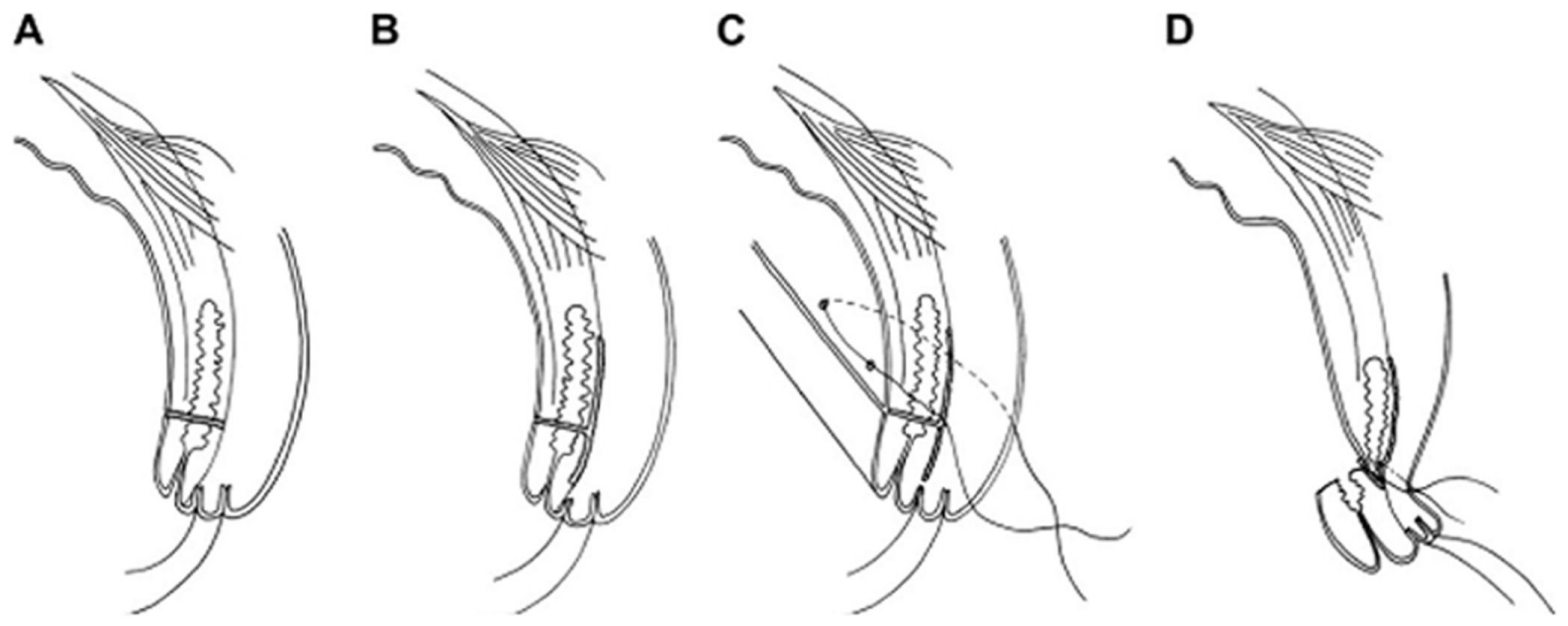
Posterior lamellar tarsal rotation (Trabut)^48^. *A*: Posterior lamellar incision. *B*: Dividing anterior and posterior lamellae. *C*: Horizontal mattress sutures. *D*: Postoperative lid eversion.

**Figure 3C:**
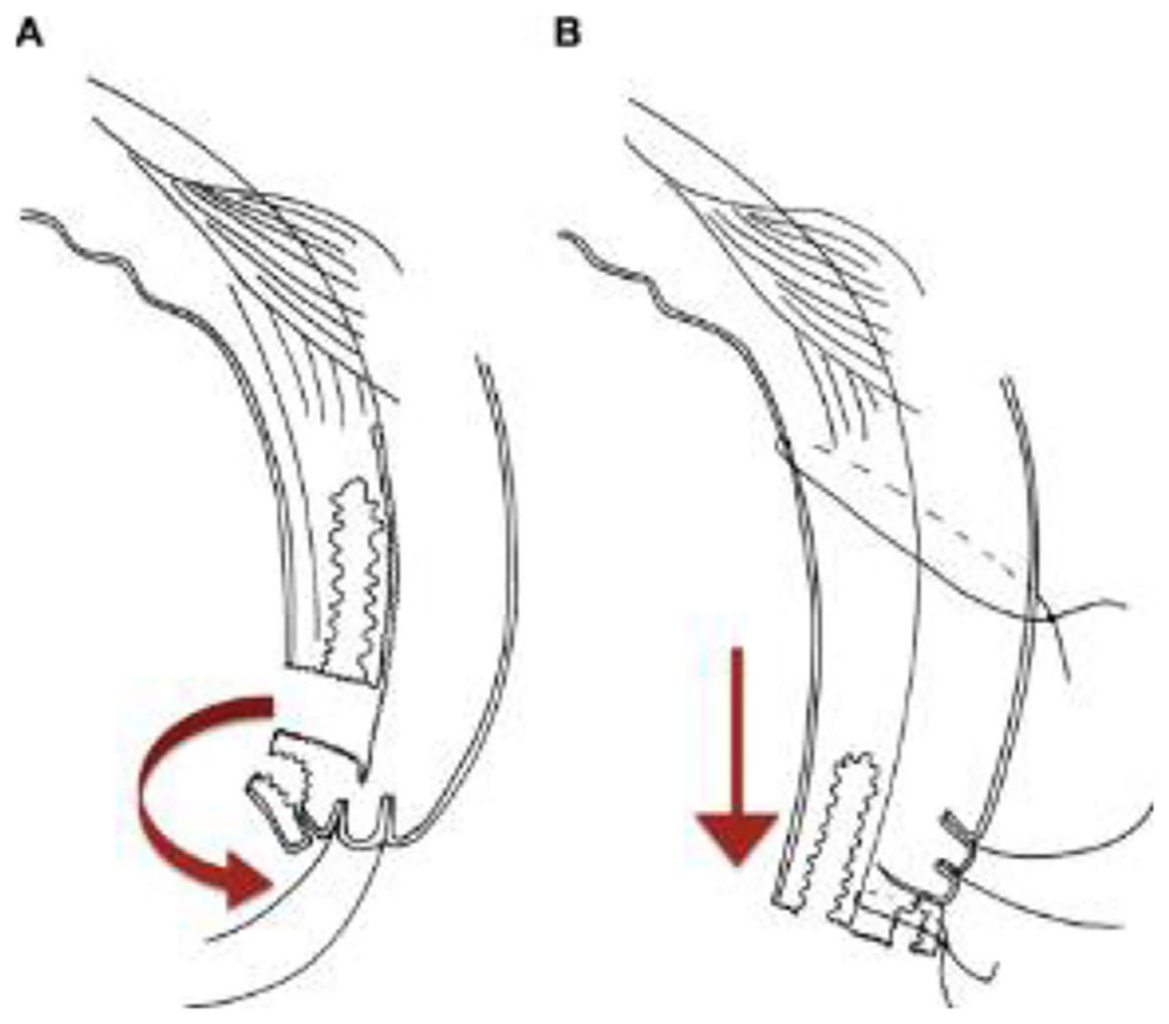
Tarsal advance and rotation^48^. *A*: Posterior lamellar incision and division between posterior and anterior lamellae (arrow indicates 180 rotation of terminal tarsus). *B*: Rotation and suturing of terminal tarsus, inferior advancement and suturing of posterior lamella (arrow indicates inferior movement of posterior lamella).

**Table 1:** Flowchart of article selection.

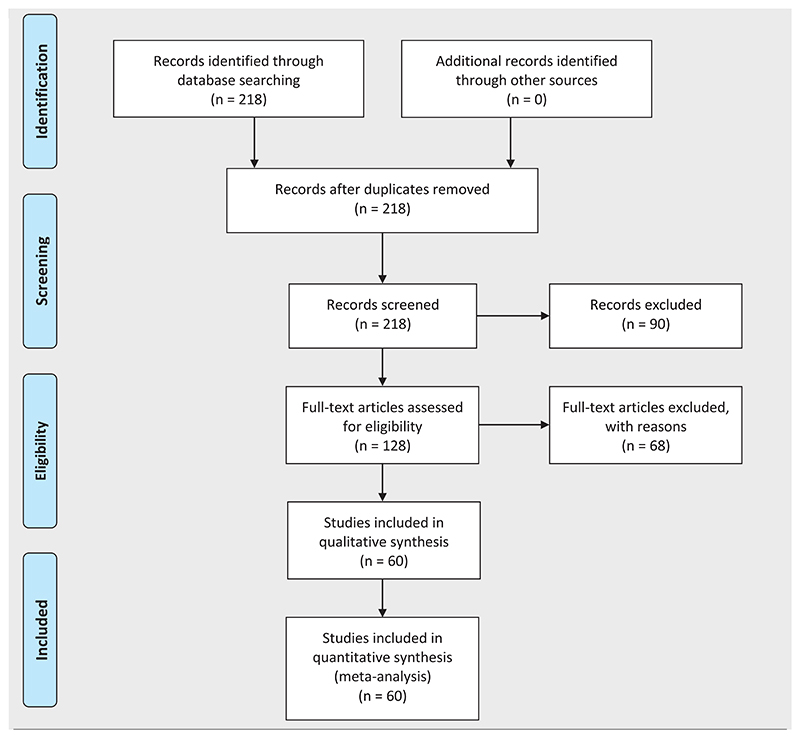

**Table 2:** Trichiasis severity classification.

Category	STAR Trial	PRET Trial
Mild	1–4 trichiatic eyelashes and no epilation OR Epilation and no trichiatic eyelashes	1–4 trichiatic eyelashes and no epilation OR <1/3 of eyelid epilated and no trichiatic eyelashes
Moderate	5–9 trichiatic eyelashes and no epilation OR 1-4 trichiatic eyelashes and epilation	5–9 trichiatic eyelashes and no epilation OR 1-4 trichiatic eyelashes and <1/3 epilated
Severe	5–9 trichiatic eyelashes and epilation OR 10+ trichiatic eyelashes	5–9 trichiatic eyelashes and any epilation OR 10+ trichiatic eyelashes OR
		>1/3 eyelid epilated

**Table 3:** Non-surgical and surgical treatments for primary TT and PTT.

Non-surgical treatments	
	Epilation (manual removal of eyelash(es), usually with forceps) Eyelid-taping (to hold eyelash(es) in the correct position)
Surgical treatments	
Surgical procedures for eyelash ablation or removal	Electrolysis (fine needle used to pass electric current to base of Eyelash follicle(s))
	Cryotherapy (freezing of the eyelash follicle(s))
	Excision of eyelash-bearing tissue
Surgical options for the treatment of upper eyelid entropion^60^	Bilamellar tarsal rotation (BLTR): full-thickness incision through the eyelid, including the scarred tarsal plate, orbicularis oculi and the skin, fixation with everting sutures Posterior lamellar tarsal rotation (PLTR)/modified Trabut: incision through the scarred tarsal plate and conjunctiva only, leaving the skin and orbicularis oculi intact, fixation with everting sutures
	Tarsal advance and rotation: incision of the tarsal plate and rotation of the terminal portion. The upper part of the tarsus is separated from the anterior lamellar, advanced and fixed with sutures
Surgical options for the treatment of upper eyelid PTT	Any of the above procedures combined, with or without blepharoplasty and/or wedge excision B-RAP^60^
	5-Step Approach
